# Resting‐state brain activity and association with baseline physical activity

**DOI:** 10.1002/alz.088841

**Published:** 2025-01-09

**Authors:** Georgia Koromila, Sofia Faraza, Martin Dyrba, Dominik Wolf, Florian U Fischer, Kristel Knaepen, Bianca Kollmann, Harald Binder, Andreas Mierau, David Riedel, Andreas Fellgiebel, Oliver Tüscher, Stefan Teipel

**Affiliations:** ^1^ Deutsches Zentrum für Neurodegenerative Erkrankungen (DZNE), Rostock Germany; ^2^ Faculty of Medicine, University of Thessaly, Larissa Greece; ^3^ University Medical Center Rostock, Rostock Germany; ^4^ Deutsches Zentrum für Neurodegenerative Erkrankungen Rostock/Greifswald (DZNE), Rostock Germany; ^5^ University Medical Center Mainz, Mainz Germany; ^6^ Center for Mental Health in Old Age, Mainz Germany; ^7^ German Sport University Cologne, Cologne Germany; ^8^ Leibniz Institute for Resilience Research, Mainz Germany; ^9^ Institute of Medical Biometry and Biostatistics, Faculty of Medicine and Medical Center, Freiburg Germany; ^10^ LUNEX International University of Sports, Exercise and Sports, Differdange Luxembourg; ^11^ Deutsches Zentrum für Neurodegenerative Erkrankungen e. V. (DZNE) Rostock/Greifswald, Rostock Germany; ^12^ Department of Psychosomatic Medicine, Rostock University Medical Center, Rostock Germany

## Abstract

**Background:**

Normal aging is associated with alterations of functional connectivity (FC) in brain neuronal networks. Altered network connectivity may be associated with accelerated cognitive decline. Physical activity is considered a beneficial lifestyle factor for maintaining cognitive health. Higher intensities of physical activity may induce structural and functional changes in the brain, particularly in regions involved in cognitive functions, such as memory, attention and executive functions. However, the underlying neural mechanisms are not widely investigated. Our aim was to examine the association between resting‐state FC of brain networks and baseline physical activity in healthy older adults.

**Method:**

We analyzed baseline resting‐state fMRI and baseline physical activity data of 149 healthy older adults (mean age: 68 years) from the AgeGain study. Physical activity was measured by using actigraphs worn for 7 days. Different intensities were measured, such as light, mean and moderate‐to‐vigorous activity (min/d). We used Independent Component Analysis (ICA) and seed‐based approaches to examine brain network activity in the Default Mode Network (DMN), Salience Network (SAL), Central Executive Network (CEN), Visual Network (VN) for cognitive effects and Sensorimotor Network (SMN) for physical effects.

**Result:**

We observed statistically significant associations between functional activation within SMN and light physical activity and spatially restricted effects for DMN and moderate‐to‐vigorous physical activity (*p* <.01 uncorrected). In addition, we observed an overlap on frontal activation across DMN, SMN and SAL. Results of the seed‐based analysis will be presented at the conference.

**Conclusion:**

Light to higher intensities of physical activity showed an association with higher functional activation of networks previously associated with cognitive decline and physical activity. This agrees with the notion that physical activity may be a protective factor against cognitive decline. Further research is needed to test the replicability of these results.

